# Methods for detecting associations between phenotype and aggregations of rare variants

**DOI:** 10.1186/1753-6561-5-S9-S51

**Published:** 2011-11-29

**Authors:** Fan Yang, Chul Joo Kang, Paul Marjoram

**Affiliations:** 1 Department of Preventive Medicine, University of Southern California, 1540 Alcazar Street, Los Angeles, CA 90089, USA

## Abstract

Although genome-wide association studies have uncovered variants associated with more than 150 traits, the percentage of phenotypic variation explained by these associations remains small. This has led to the search for the dark matter that explains this missing genetic component of heritability. One potential explanation for dark matter is rare variants, and several statistics have been devised to detect associations resulting from aggregations of rare variants in relatively short regions of interest, such as candidate genes. In this paper we investigate the feasibility of extending this approach in an agnostic way, in which we consider all variants within a much broader region of interest, such as an entire chromosome or even the entire exome. Our method searches for subsets of variant sites using either Markov chain Monte Carlo or genetic algorithms. The analysis was performed with knowledge of the Genetic Analysis Workshop 17 answers.

## Background

The modern genomics era holds forth promise of great advances in terms of our understanding of the genetic causes of disease. The ability to interrogate orders of magnitude more data than what was previously available has indeed led to a rapid increase in the rate at which we uncover variants, such as single-nucleotide polymorphisms (SNPs), that are associated with phenotypes. For example, a meta-analysis of height has led to the discovery of about 50 associated variants (see [1] and refs therein). However, the total variance explained by those variants is 5%, whereas from empirical observation we know that heritability of height is about 80%. This somewhat depressing scenario has been repeated in many disease studies (in which the sample sizes are typically much smaller than was the case for height) and has led to the adoption of the term *dark matter* to describe this phenomenon [2].

Dark matter is the purported explanation for the missing genetic heritability of phenotype. One potential form of dark matter is rare variants [3]. Modern so-called SNP-chip platforms (e.g., Illumina or Affymetrix technologies) were designed, with good reason, to use only common SNPs. This design is likely to give the greatest cost efficiency in terms of gathering as much data as possible given the physical and/or financial restraints in play. However, it also means that if disease phenotypes are in large part due to the presence of collections of rare variants, the so-called multiple rare variants (MRV) hypothesis, then such platforms are likely to have low utility for detecting these associations. Of course, it is also possible that dark matter is in fact just lack of power (e.g., as a result of our poor ability to detect interactions), but in this paper we focus on the first possible explanation by analyzing the Genetic Analysis Workshop 17 (GAW17) data [4] and searching for MRVs.

We focus on methods in which scores are calculated for aggregations of SNPs. Several score statistics have been proposed (see Methods section), but all have been applied in contexts in which a relatively small region of interest (say, a gene) is under consideration. In this application we seek to extend the application of such statistics to larger contexts. We do this by applying optimization algorithms to detect appropriate subsets of the set of all possible SNPs. We then apply these methods to the GAW17 data, for the quantitative trait data, assessing significance of results by means of permutation tests.

## Methods

### Score statistics

We focus on three score statistics. First, we consider the statistic *σ*_LL_, motivated by Li and Leal [5], in which the score of a set of SNPs **S** = {*S*_1_, …, *S_n_*} for individual *I* is defined to be the total count of mutant alleles summed across those SNPs for *I*. Second, we consider the statistic *σ*_MB_, which follows Madsen and Browning [6] in that the contribution to the score from each SNP is weighted by the inverse of the frequency of its minor allele in the sample. Third, we consider a novel statistic, *σ*_ME_, which weights the count of mutant alleles for a SNP by the marginal effect size of that SNP, estimated either by log odds ratio for a binary trait or by the coefficient in the univariate linear regression for a quantitative trait.

### Optimization algorithms

Traditionally, score statistics are applied to predetermined small regions of interest, and all SNPs contained in that region are included in the evaluation of the score statistic. It would be useful to be able to extend the region of search so that the analysis might be more agnostic regarding what is being searched for. For example, we might consider a region defined as all genes in a given pathway. To explore the computational limits to this approach, we use a chromosome as a unit corresponding to a “large region.” Unfortunately, the performance of score statistic methods, such as those mentioned, is known to deteriorate as the fraction of nonassociated statistics in the region increases. For that reason, to extend these methods to wider regions, we use an optimization method to choose a subset of statistics for which the score is calculated. For the purposes of the present study and after some experimentation, we choose to use subsets of fixed size: 20 SNPs. We compare results for two optimization methods: the Markov chain Monte Carlo (MCMC) method and genetic algorithms (GAs).

In our MCMC implementation we run five chains in parallel (Metropolis coupled MCMC). The MCMC process depends crucially on its proposal kernel, the rule by which SNPs are added or removed from the set of SNPs currently under consideration. Here, we use a kernel that samples SNPs with a probability proportional to their association with the phenotype of interest. We use the square of the Pearson correlation coefficient as the measure of association between score statistic and phenotype. For mathematical convenience, we assume that the residuals of the three quantitative trait loci (Q1, Q2, and Q4) are normally distributed after model fitting and that there is a linear relationship between the value of the score statistic and the mean trait value. This allows us to approximate the likelihood term contained within the Hastings ratio as a function of the correlation coefficient between score and trait; otherwise, the likelihood term would be intractable.

Each run of the MCMC process results in a posterior distribution for the inclusion of each SNP in the subset of SNPs that is used to calculate the score. Therefore these SNPs may be associated with the phenotype of interest. To make this more directly comparable with GAs, which search for a single, optimal subset, we also record the single best solution discovered in each run. This also allows for simple compilation of results across replicates when we report summaries of our results.

For GAs, populations of “chromosomes” evolve in a context in which their fitness is measured by their ability to predict the phenotype of interest. Here, the chromosome simply defines a subset of SNPs, and fitness is measured as a function of the Pearson correlation coefficient between the score and the quantitative trait for those SNPs. The chromosomes evolve subject to mutation and recombination; the identity of the SNPs contained in each chromosome (and hence used when the score is calculated) changes. Probability of reproduction is proportional to fitness, thus encouraging the more successful chromosomes to have more “offspring.” A full description of GAs is not possible given the space constraints here, but see Mitchell [7] for a more comprehensive introduction.

### Assessment of significance

In all cases we assess significance of overall fit by creating 1,000 data sets in which the phenotype is randomly permuted to determine the null distribution of the best-performing score statistic at the end of the analysis. To assess whether we are finding the functional SNPs or regions, we also report the frequency with which SNPs were included in the optimal solution, accumulated across all 200 phenotype replicates.

## Results

### Inflated type I error rates

We begin by running our analyses on entire chromosomes, one chromosome at a time. Empirical *p*-values for overall model fit are obtained as described in the “Assessment of Significance” subsection and are shown in Table 1 for the MCMC analysis (see the column for unadjusted results), and in Table 2 for the GA analysis (again, see the unadjusted column in the table). For convenience, results for chromosomes containing SNPs that are directly associated with the corresponding trait (per the GAW17 answers) are shown in boldfaced text. The results are similar for all three statistics we considered, so here and throughout the manuscript where results for just one statistic are presented, we show results for the Madsen-Browning statistic (*σ*_MB_) only.

**Table 1:** MCMC analysis: chromosome-wide *p*-values

	Q1		Q2		Q4	
	
Chromosome	Unadjusted	Adjusted	Unadjusted	Adjusted	Unadjusted	Adjusted
1	**0.020**	**0.148**	0.688	0.594	0.061	0.213
2	0.001	0.285	**0.217**	**0.348**	0.542	0.403
3	0.026	0.240	**0.117**	**0.811**	0.810	0.593
4	**0.002**	**0.414**	0.191	0.183	0.202	0.641
5	**0.008**	**0.841**	0.050	0.658	0.544	0.038
6	**0.002**	**0.014**	**0.187**	**0.050**	0.002	0.022
7	0.001	0.017	**0.003**	**0.459**	0.507	0.333
8	0.044	0.164	**0.068**	**0.939**	0.351	0.875
9	0.022	0.232	**0.174**	**0.869**	0.001	0.124
10	0.002	0.844	**0.183**	**0.390**	0.586	0.532
11	0.002	0.223	0.601	0.874	0.266	0.357
12	0.010	0.267	**0.310**	**0.692**	0.551	0.235
13	**0.001**	**0.001**	0.097	0.047	0.266	0.707
14	**0.001**	**0.006**	0.64	0.832	0.551	0.808
15	0.140	0.563	0.923	0.663	0.006	0.571
16	0.016	0.229	0.198	0.190	0.269	0.245
17	0.008	0.464	**0.499**	**0.694**	0.355	0.680
18	0.004	0.037	0.945	0.958	0.033	0.046
19	**0.001**	**0.034**	0.519	0.368	0.514	0.570
20	0.904	0.003	0.053	0.586	0.034	0.224
21	0.597	0.611	0.076	0.552	0.015	0.064
22	0.002	0.633	0.170	0.831	0.008	0.628

Chromosome-level *p*-values for each quantitative trait using the Madsen-Browning statistic, replicate 1. Results are shown before (unadjusted) and after (corrected) PC correction for population stratification. Results for chromosomes containing SNPs that are directly associated with the corresponding trait (per the GAW17 answers) are shown in boldface.

**Table 2:** GA analysis: chromosome-wide *p*-values

	Q1		Q2		Q4	
	
Chromosome	Unadjusted	Adjusted	Unadjusted	Adjusted	Unadjusted	Adjusted
1	**0.018**	**0.506**	0.385	0.632	0.149	0.015
2	0.001	0.001	**0.311**	**0.401**	0.137	0.769
3	0.017	0.912	**0.235**	**0.958**	0.364	0.371
4	**0.006**	**0.021**	0.054	0.726	0.136	0.089
5	**0.002**	**0.128**	0.041	0.947	0.082	0.067
6	**0.001**	**0.003**	**0.169**	**0.209**	0.019	0.013
7	0.002	0.070	**0.067**	**0.665**	0.017	0.422
8	0.258	0.140	**0.646**	**0.473**	0.078	0.220
9	0.017	0.124	**0.124**	**0.103**	0.065	0.061
10	0.196	0.003	**0.174**	**0.434**	0.042	0.362
11	0.006	0.032	0.609	0.869	0.190	0.702
12	0.017	0.073	**0.393**	**0.730**	0.167	0.178
13	**0.001**	**0.002**	0.482	0.568	0.427	0.060
14	**0.002**	**0.001**	0.958	0.404	0.295	0.160
15	0.541	0.266	0.422	0.269	0.254	0.579
16	0.156	0.401	0.280	0.364	0.235	0.643
17	0.588	0.403	**0.619**	**0.966**	0.509	0.598
18	0.012	0.056	0.417	0.840	0.464	0.670
19	**0.067**	**0.014**	0.262	0.554	0.041	0.479
20	0.152	0.006	0.048	0.204	0.054	0.136
21	0.663	0.073	0.006	0.250	0.088	0.065
22	0.004	0.062	0.127	0.108	0.072	0.095

Chromosome-level *p*-values for each quantitative trait using the Madsen-Browning statistic, replicate 1. Results are shown before (unadjusted) and after (corrected) PC correction for population stratification. Results for chromosomes containing SNPs that are directly associated with the corresponding trait (per the GAW17 answers) are shown in boldface.

**Figure 1:**
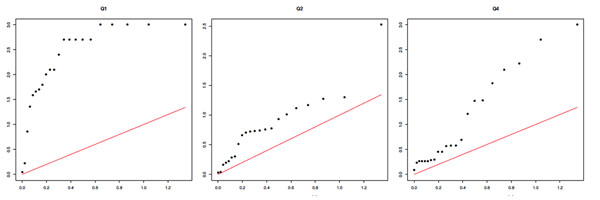
**MCMC results: Q-Q plots of chromosome-wide *p*-values for replicate 1***. x*-axis: expected −log_10_*p*. *y*-axis: observed −log_10_*p*.

**Figure 2:**
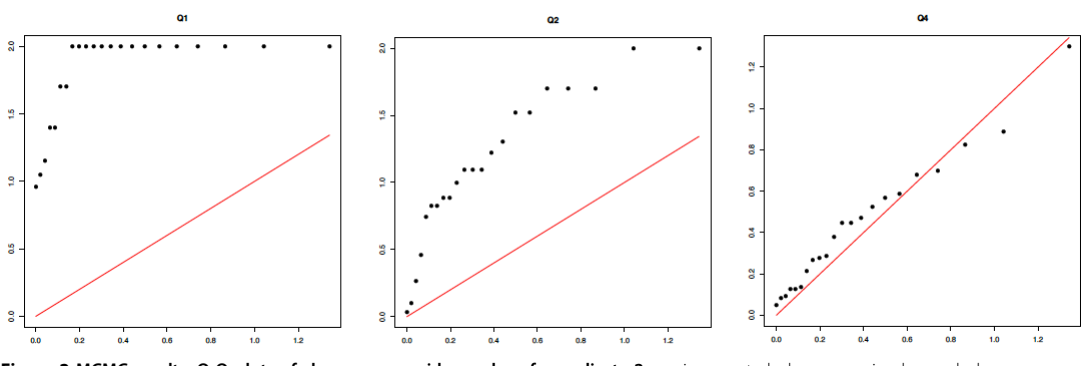
**MCMC results: Q-Q plots of chromosome-wide *p*-values for replicate 2. ***x*-axis: expected −log_10_*p*. *y*-axis: observed −log_10_*p*.

The most striking feature of the results is a grossly inflated type I error rate. To further demonstrate this issue and to demonstrate the degree to which it is reproducible across replicates, we present quantile-quantile (Q-Q) plots for replicates 1 and 2 for results from the MCMC analysis in Figures 1 and 2. (Results from the GA analysis were similar and so are not shown.) We see a clear inflation of type I error across both replicates, most strikingly for Q1 but also for Q2 and, in replicate 1 only, Q4, which has no direct association with any SNP in this data set. The difference between the results for replicates 1 and 2 for Q4 is striking because this is essentially stochastic noise (the replicates are independent, identically distributed realizations of the same disease model simulation *with exactly the same SNP data*).

**Figure 3:**
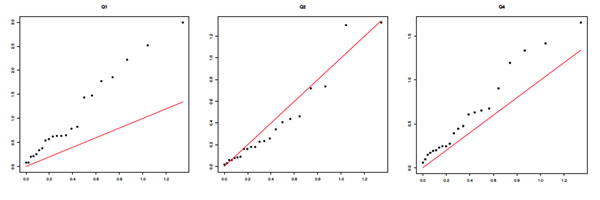
**MCMC results: Q-Q plots of chromosome-wide *p*-values for replicate 1 after correction for population structure using 10 PCs. ***x*-axis: expected −log_10_*p*. *y*-axis: observed −log_10_*p*.

These results clearly indicate the need for correction for inflated type I error, which is likely partly the result of population stratification. For this reason, we adjust for population structure using principal components (PCs). After exploring a range of the possible number of PCs to include in the analysis, we determined that 10 PCs perform well in terms of reducing the inflation of type I error, and so all further results in this paper show results obtained after regressing out the first 10 PCs. Overall *p*-values for model fit across each chromosome are shown in Table 1 (see the adjusted column), and we show Q-Q plots for the PC-adjusted MCMC results in Figure 3. Although the Q-Q plots for replicate 1 now look more satisfactory, with the null trait, Q4, looking suitably uniform (although Q1 is still highly inflated), we note that these results vary a good deal across replicates. For example, Q2 shows some inflation of −log *p*-values after PC correction in replicate 2 (see Figure 2), and because Q2 should show association with some of the SNPs in the data, this may well prove to be a better outcome than what we see in replicate 1, which most likely indicates a loss of power. However, because we wish to avoid mining the data—choosing a different number of PCs for each replicate (this is computationally intractable in the time available anyway)—for the results shown we analyze each of the 200 replicates after adjusting for population stratification using 10 PCs.

### What SNPs are detected?

Tables 1 and 2 report *p*-values for the overall fit of the model on each chromosome, but they do not indicate whether we are actually finding the causal SNPs. To assess this, in Tables 3 and 4 we consider the results of an independent analysis of each of the 200 replicates of the GAW17 data and report the *p*-values resulting from a chi-square test for nonrandom association between those SNPs that were included in the best aggregation of SNPs found by our MCMC (Table 3) and GA (Table 4) analysis (20 SNPs for each replicate) and those that were actually causal (assessed by referencing the GAW17 answers). Analysis was performed after correction for population stratification using 10 PCs. Of course, we cannot expect to necessarily detect a causal SNP, because any other SNP in perfect linkage disequilibrium will serve just as well in our score statistic and other SNPs in high linkage disequilibrium might also be detected instead. Despite these practical realities, we see that, although results do vary from chromosome to chromosome, we do frequently (but not always) observe a significant tendency to preferentially include causal SNPs for analyses in which significance was observed at the chromosomal level (cf. *p*-values in Tables 1 and 2).

**Table 3:** MCMC analysis: chi-square test for detection of causal SNPs

Q1		Q2	
Chromosome	*p*-value	Chromosome	*p*-value

1	1.1 × 10^−2^	2	0
4	1.5 × 10^−8^	3	3.5 × 10^−3^
5	1.7 × 10^−1^	6	0
6	0	7	4.2 × 10^−2^
13	0	8	7.7 × 10^−7^
14	2.0 × 10^−1^	9	4.6 × 10^−1^
19	8.5 × 10^−1^	10	1.2 × 10^−2^
		12	2.4 × 10^−1^
		17	4.2 × 10^−1^

We report *p*-values resulting from a chi-square test for nonrandom association, across the 200 replicates of the GAW17 data, between those SNPs that were included in the best aggregation of SNPs found by our MCMC analysis and those that were actually causal (assessed by referencing the GAW17 answers). Analysis was performed after correction for population stratification using 10 PCs.

**Table 4:** GA analysis: chi-square test for detection of causal SNPs

Q1		Q2	
Chromosome	*p*-value	Chromosome	*p*-value

1	6.2 × 10^−2^	2	3.4 × 10^−1^
4	0	3	0
5	0	6	0
6	9.3 × 10^−2^	7	2.8 × 10^−3^
13	0	8	0
14	0^a^	9	3.1 × 10^−1^
19	2.5 × 10^−4^	10	0
		12	5.2 × 10^−2^
		17	1.3 × 10^−1^

We report *p*-values resulting from a chi-square test for nonrandom association, across the 200 replicates of the GAW17 data, between those SNPs that were included in the best aggregation of SNPs found by our GA analysis and those that were actually causal (assessed by referencing the GAW17 answers). Analysis was performed after correction for population stratification using 10 PCs.

^a^ For the Q1–chromosome 14 combination, there was a significant tendency to miss the causal SNPs.

### Illustrative results

**Figure 4:**
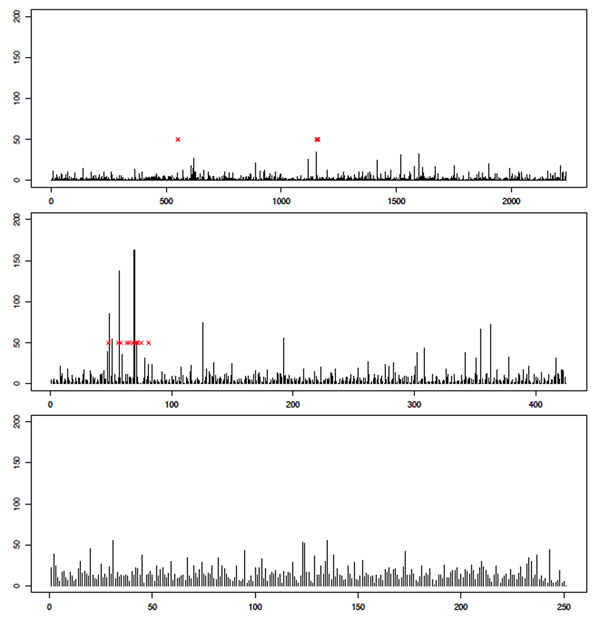
**MCMC results for analysis of quantitative trait Q1**. Histogram showing the frequency with which each SNP was included in the subset of SNPs on which the optimal score statistic was calculated, aggregated across all 200 replicates. Results are for chromosome 1 (top), chromosome 13 (middle), and chromosome 21 (bottom). The *x*-axis indexes the SNPs on the given chromosome (each bar corresponds to a single SNP; bars are equally spaced for clarity). The *y*-axis shows the frequency with which the SNP was included in the optimal solution. Red x’s indicate positions of SNPs that are associated with the trait (according to GAW17 answers).

**Figure 5:**
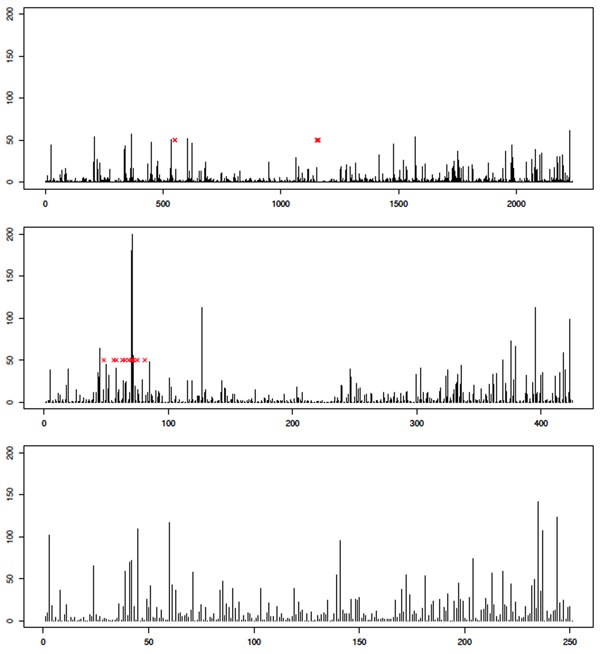
**GA results for analysis of quantitative trait Q1**. Histogram showing the frequency with which each SNP was included in the subset of SNPs on which the optimal score statistic was calculated, aggregated across all 200 replicates. Results are for chromosome 1 (top), chromosome 13 (middle), and chromosome 21 (bottom). The *x*-axis indexes the SNPs on the given chromosome (each bar corresponds to a single SNP; bars are equally spaced for clarity). The *y*-axis shows the frequency with which the SNP was included in the optimal solution. Red x’s indicate positions of SNPs that are associated with the trait (according to GAW17 answers).

Having investigated the issue of type I error rates and having made some attempt to mitigate its effects, we now show illustrative results for particular chromosomes of interest. Space prohibits including a large number of examples, so we instead provide results for analysis of Q1 on three chromosomes: 1, 13, and 21. Chromosome 13 contains a region with large effect, so we might hope to see a strong signal. Chromosome 1 contains two SNPs affecting Q1, and chromosome 21 contains no associated SNPs. Results for the MCMC analysis are shown in Figure 4 and those for the GA analysis are shown in Figure 5. We see qualitatively different behavior, with a clear peak being seen on chromosome 13 but not on chromosome 1 or 21. Furthermore, the signal appears to be stronger in the MCMC analysis than in the GA analysis. This trend is observed across most analyses. However, it is important to note that in the analysis in which we correct for population structure, chromosome 1 does not attain a significant *p*-value after the permutation test, whereas without PC correction for structure, significance is obtained in most cases. This illustrates a peril of correcting for population structure: If phenotype is also correlated with structure, then loss of power can result.

**Figure 6:**
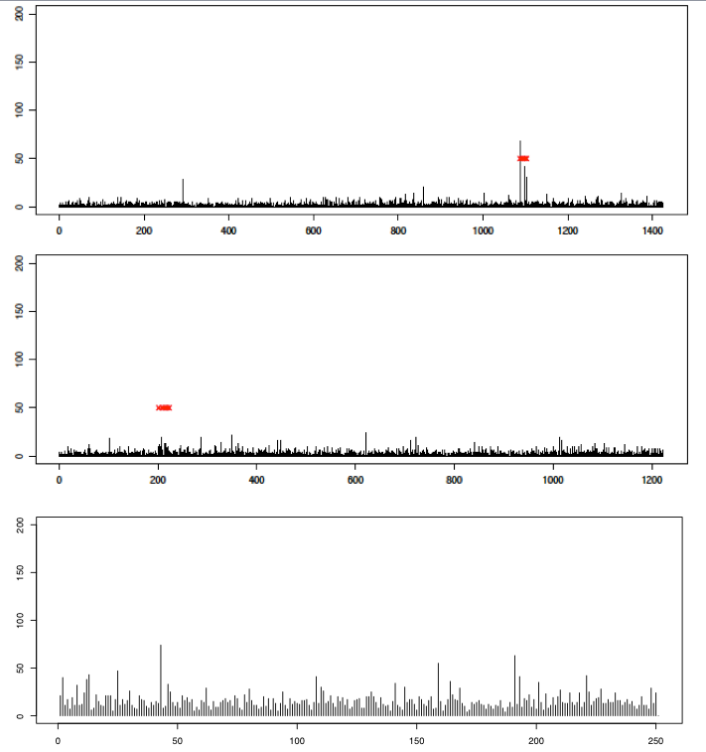
**MCMC results for analysis of quantitative trait Q2**. Histogram showing the frequency with which each SNP was included in the subset of SNPs on which the optimal score statistic was calculated, aggregated across all 200 replicates. Results are for chromosome 6 (top), chromosome 17 (middle), and chromosome 21 (bottom). The *x*-axis indexes the SNPs on the given chromosome (each bar corresponds to a single SNP; bars are equally spaced for clarity). The *y*-axis shows the frequency with which the SNP was included in the optimal solution. Red x’s indicate positions of SNPs that are associated with the trait (according to GAW17 answers).

**Figure 7:**
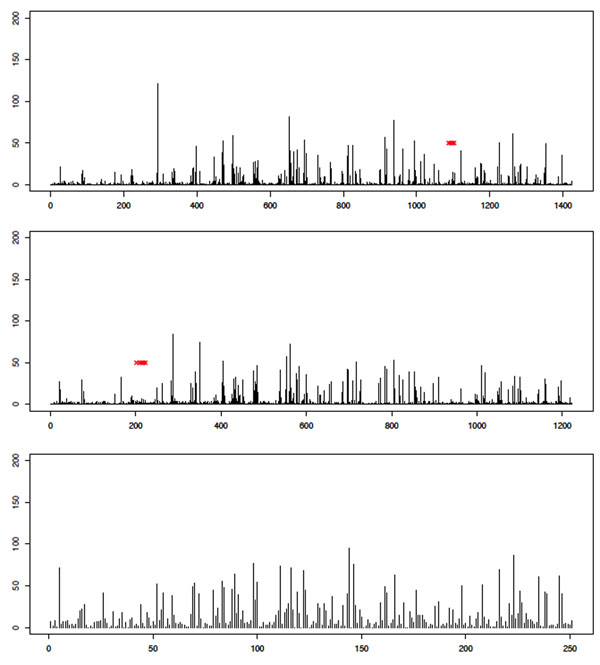
**GA results for analysis of quantitative trait Q2.** Histogram showing the frequency with which each SNP was included in the subset of SNPs on which the optimal score statistic was calculated, aggregated across all 200 replicates. Results are for chromosome 6 (top), chromosome 17 (middle), and chromosome 21 (bottom). The *x*-axis indexes the SNPs on the given chromosome (each bar corresponds to a single SNP; bars are equally spaced for clarity). The *y*-axis shows the frequency with which the SNP was included in the optimal solution. Red x’s indicate positions of SNPs that are associated with the trait (according to GAW17 answers).

As a further example we show the results of the analysis of Q2 on chromosomes 6, 17, and 21. Again these chromosomes are chosen because they should have strong, medium, and no signal, respectively. The results are shown in Figures 6 and 7. Again, power appears to be lost because of the correction for stratification, although the MCMC analysis does appear to find a clear signal for chromosome 6. Interestingly the GA analysis results for chromosome 6 before PC correction also show a clear peak (results not shown).

**Figure 8:**
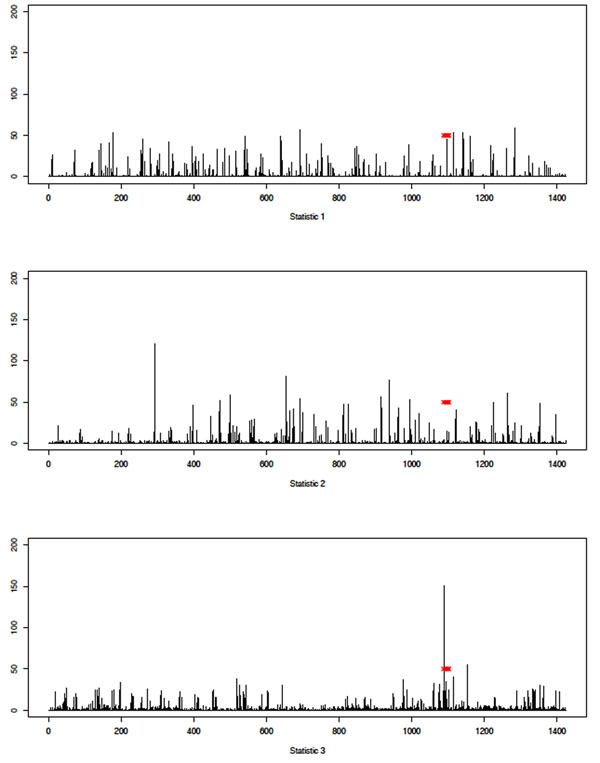
**Example of differing results from the three statistics**. Histograms showing the frequency with which each SNP was included in the subset of SNPs on which the optimal score statistic was calculated, aggregated across all 200 replicates. Results are for the GA analysis of Q2 on chromosome 6. Results are for *σ*_LL_ (top), *σ*_MB_ (middle), and *σ*_ME_ (bottom). The *x*-axis indexes the SNPs on chromosome 6 (each bar corresponds to a single SNP; bars are equally spaced for clarity). The *y*-axis shows the frequency with which the SNP was included in the optimal solution. Red x’s indicate positions of SNPs that are associated with the trait (according to GAW17 answers).

Finally, we present an illustrative but representative example of the differences observed between the performances of the three statistics. This is shown in Figure 8, where we see results of the GA analysis of Q2 on chromosome 6. We comment further on this in the Discussion and conclusions section.

## Discussion and conclusions

Recently, many methods have been developed that exploit score statistics to detect associations between aggregations of (rare) variants and phenotype in an attempt to uncover at least some of the causes of the phenomenon of dark matter. However, such methods have thus far been applied in contexts in which only a relatively narrow region of interest is being investigated and, typically, all SNPs contribute to the score (subject to inclusion criteria such as thresholds based on minor allele frequency, for example). It would be of great use to have similar approaches that are more agnostic in nature and that explore entire chromosomes, or ideally genomes, to detect subsets of SNPs for which a significant association between phenotype and score is obtained when a score is calculated in a manner analogous to the existing methods for narrow regions.

The problem, of course, is the sheer magnitude of the number of possible subsets one might consider. It is impossible to explore the space of all possible subsets exhaustively. For that reason, some sort of guidance is needed. Here, we use two search algorithms: GAs, which are designed to find optimal solutions in a complex state space; and MCMC, which is designed to calculate posterior distributions defined over complex state spaces.

The GAW17 data set proved troublesome for all groups to analyze, in large part because of the extremely high type I errors. To some degree this remained an issue even after adjustment for population structure, as is the case in our own analysis. As such, power is low across the board, and this means that it is hard to draw meaningful conclusions regarding the relative merits of alternative methods. This is the case with our own analysis. Optimization with the MCMC method appears to perform better than that with GAs in general, but it is not clear whether this might be due to the particular way in which the GA was implemented (perhaps longer runs or use of different evolutionary parameters might improve performance) or whether it is a consequence of particular features of the data.

Our experience in the current setting is that attempting to search across the entire genome or the portion of the exome included in the GAW17 data has proved unsuccessful. Even with the reduced size of the GAW17 data compared to what would be obtained in a full exome- or genome-wide next-generation sequencing study and given the consequent lower dimension of the state space over which the selection of SNPs needs to be optimized, in general we could not find meaningful optima [results not shown]. For that reason we focus in this paper on results for chromosome-wide analysis.

When we use the chromosome as the unit of analysis, the results are encouraging in the sense that we tend to obtain more significant results on chromosomes on which associated SNPs are found. However, once a chromosome has been identified as containing an association with phenotype, the key question becomes which (set of) SNPs are driving the association? As we have shown in the illustrative results in this paper, although sometimes clear signals are found (e.g., for Q1 on chromosome 13), in other cases there is no clear signal to show which SNPs are associated (e.g., for Q2 on chromosome 17). This problem was likely worsened by the problems with type I error, which drastically curtailed overall power. However, in Tables 3 and 4 we show a significant tendency to pick out the truly associated SNPs in cases in which the chromosome-wide *p*-value is small and sometimes even when significance was not obtained at the chromosomal level.

As the size of region being considered grows larger, it becomes imperative to find some other way of guiding the search for optimal subsets of SNPs. One potential approach is to include external information regarding which SNPs are most likely to be informative. For example, one might include functional information from external databases. This should improve the performance of the search algorithms and thereby allow for more successful optimization over larger regions.

Another issue with methods that search across a large state space of potentially informative variation is the issue of overfitting. In this paper we guard against this in two ways: by using fixed-size subsets of SNPs and by assessing significance by means of a permutation test. A further response to this issue would be to use shrinkage methods, such as penalized regression, to reduce the number of variables, in our case SNPs, that might be included in the optimal subset. We intend to explore this approach in future work.

Finally, we see some evidence that the novel score statistic we propose in this paper may prove to be more powerful than existing statistics (see Figure 8). Rather than concluding anything regarding the relative merits of the statistics considered here, which is impossible given the focus on the GAW17 data in particular, we note that optimal construction of a score statistic is still an open question and is worthy of further investigation.

In summary, although our results are somewhat preliminary, in that the conclusions are based on a single data set, at least in terms of the genotype data, we believe that they do show the potential of using optimization approaches to extend existing score-based methods over wider regions than those currently considered. Selection of a subset of SNPs within a region should lessen the existing tendency for the performance of such methods to deteriorate as the number of nonassociated SNPs in the region of interest increases. In traditional analyses this occurs because such SNPs are always included in the calculation of the score statistic, whereas by choosing a subset of SNPs, such statistics will be excluded, if the method is working well, leading to an expected increase in power.

## Competing interests

The authors declare that there are no competing interests.

## Authors’ contributions

FY and CJK participated in the design of the study and performed the simulations and statistical analysis. PM conceived of the study and wrote the manuscript. All authors read and approved the final manuscript.
